# Participation of lipopolysaccharide in hyperplasic adipose expansion: Involvement of NADPH oxidase/ROS/p42/p44 MAPK‐dependent Cyclooxygenase‐2

**DOI:** 10.1111/jcmm.17419

**Published:** 2022-06-01

**Authors:** Chao‐Chien Chang, Kee‐Chin Sia, Jia‐Feng Chang, Chia‐Mo Lin, Chuen‐Mao Yang, I‐Ta Lee, Thi Thuy Tien Vo, Kuo‐Yang Huang, Wei‐Ning Lin

**Affiliations:** ^1^ Department of Cardiology, Cathay General Hospital Taipei Taiwan; ^2^ Department of Pharmacology, School of Medicine, College of Medicine Taipei Medical University Taipei Taiwan; ^3^ School of Medicine Fu Jen Catholic University New Taipei City Taiwan; ^4^ Graduate Institute of Biomedical and Pharmaceutical Science Fu Jen Catholic University New Taipei City Taiwan; ^5^ Department of Internal Medicine, En‐Chu‐Kong Hospital New Taipei City Taiwan; ^6^ Department of Nursing Yuanpei University of Medical Technology Hsinchu Taiwan; ^7^ Division of Chest Medicine, Shin Kong Hospital Taipei Taiwan; ^8^ Department of Chemistry Fu‐Jen Catholic University New Taipei Taiwan; ^9^ Department of Pharmacology, College of Medicine China Medical University Taichung Taiwan; ^10^ Ph.D. Program for Biotech Pharmaceutical Industry China Medical University Taichung Taiwan; ^11^ Department of Post‐Baccalaureate Veterinary Medicine, College of Medical and Health Science Asia University Taichung Taiwan; ^12^ School of Dentistry, College of Oral Medicine Taipei Medical University Taipei Taiwan; ^13^ National Defense Medical Center Graduate Institute of Pathology and Parasitology Taipei Taiwan

**Keywords:** adipose tissue, COX‐2, lipopolysaccharide, ROS

## Abstract

Obesity is a world‐wide problem, especially the child obesity, with the complication of various metabolic diseases. Child obesity can be developed as early as the age between 2 and 6. The expansion of fat mass in child age includes both hyperplasia and hypertrophy of adipose tissue, suggesting the importance of proliferation and adipogenesis of preadipocytes. The changed composition of gut microbiota is associated with obesity, revealing the roles of lipopolysaccharide (LPS) on manipulating adipose tissue development. Studies suggest that LPS enters the circulation and acts as a pro‐inflammatory regulator to facilitate pathologies. Nevertheless, the underlying mechanisms behind LPS‐modulated obesity are yet clearly elucidated. This study showed that LPS enhanced the expression of cyclooxygenase‐2 (COX‐2), an inflammatory regulator of obesity, in preadipocytes. Pretreating preadipocytes with the scavenger of reactive oxygen species (ROS) or the inhibitors of NADPH oxidase or p42/p44 MAPK markedly decreased LPS‐stimulated gene expression of COX‐2 together with the phosphorylation of p47^phox^ and p42/p44 MAPK, separately. LPS activated p42/p44 MAPK via NADPH oxidase‐dependent ROS accumulation in preadipocytes. Reduction of intracellular ROS or attenuation of p42/p44 MAPK activation both reduced LPS‐mediated COX‐2 expression and preadipocyte proliferation. Moreover, LPS‐induced preadipocyte proliferation and adipogenesis were abolished by the inhibition of COX‐2 or PEG_2_ receptors. Taken together, our results suggested that LPS enhanced the proliferation and adipogenesis of preadipocytes via NADPH oxidase/ROS/p42/p44 MAPK‐dependent COX‐2 expression.

## 1 INTRODUCTION

Obesity is a world‐wide problem, with the complication of various metabolic diseases. The problems of children with overweight and obesity are more and more serious, with over 41 million children under the age of 5 diagnosed with overweight or obese.^1^ It is found that obesity can be developed as early as child age between 2 and 6.^2^ The expansion of fat mass in child age includes both hyperplasia and hypertrophy of adipose tissue, suggesting the importance of proliferation and adipogenesis of preadipocytes.^3^ Though the hypertrophy of fat cells is reported as the characteristic of obese during the development, the hyperplasia of adipose tissue gains more and more notice. Because of the less expansion ability of fat cells, preadipocytes play the major roles in the increase of fat cell numbers.^4^ It is reported that the number of adipocytes is mostly stable through adulthood once determined in childhood and adolescence.^5^ However, recent studies in rodents have demonstrated that new adipocytes can originate from preadipocyte differentiation, which in turn can result in the expansion of adipose tissue during prolonged caloric excess.^6^, ^7^ It is found that the hyperplasia of adipose tissue occurs in both genetic and diet‐induced obese model and correlates with the disease severity and the poorest prognosis.^8^ Furthermore, there is an association between gut microbiota alteration and obesity development. The increase of *Firmicutes* and the decrease of *Bacterioidetes* and *Bifidobacterium* in obesity result in the decreased tight junction integrity and the increased gut permeability via the dysregulation of proglucagon‐derived peptide (GLP)‐2 expression.^9^ Lipopolysaccharide (LPS) is the major constituent that constitutes the cell wall of Gram‐negative bacteria. Comparing to the levels in the blood, the concentration of LPS is much higher in the gut.^10^ Studies suggest that LPS enters the circulation via directly transcellular/paracellular transport through enteric barrier, or via the internalization and incorporation of chylomicrons.^11^, ^12^ Once entering the circulation, LPS may exert its function as a pro‐inflammatory regulator to promote the M1 phenotype changes of monocytes/macrophages.^13^ In addition, LPS may modulate and expand the adipose tissues. In fact, it has been found that *Staphylococcus aureus* infection leads to the expansion of human dermal fat layer.^14^

Cyclooxygenase‐2 (COX‐2) plays important roles in regulating eicosanoids production via promoting the oxidation of arachidonic acid (AA). Upregulation of COX‐2 gene can facilitate the proliferation of cancer cells or stem cells. It is found that the expression of COX‐2 increases the stem‐like cells (SLC) in human breast cancer by prostaglandin‐dependent activation of NOTCH/WNT via PI3K/AKT signalling pathway.^15^ In addition, upregulated COX‐2 promotes bone morphogenetic protein 9 (BMP9)‐induced osteogenic differentiation of mesenchymal stem cells.^16^ COX‐2 is involved in glucose‐related proliferative retinopathy via hyperosmolarity‐mediated angiogenesis.^17^ Overexpression of COX‐2 can reverse long non‐coding RNA GAS5‐suppressed LPS‐induced human prostate epithelial cell line (RWPE‐1) and primary human prostate epithelial cells (HPECs) cell proliferation, suggesting the importance of COX‐2 in LPS effects on cell proliferation and prostatitis.^18^

Reactive oxygen species (ROS) are found to involve in various pathological conditions and connected to the detrimental complications. More and more studies from humans and experimental animal models have indicated that cardiovascular diseases together with vascular inflammation, oxidative stress, and arterial remodelling are linked with the dysfunction of perivascular adipose tissue.^19^ It is found that oxidative stress‐related DNA damage is high in obese subjects and bariatric surgery can induce weight loss together with DNA strand break reduction.^20^ ROS are also reported to involve in accelerating adipogenesis of human adipose‐derived stem cells by promoting differentiation.^21^ Activation of NADPH oxidase is one of the sources of ROS production in adipose tissue.^19^ Knockout of p47^phox^, an essential cofactor required for NADPH oxidase 2, can attenuate high fat‐induced obesity in female mice.^22^ Moreover, in our previous works, NADPH oxidase‐mediated ROS could contribute to COX‐2 expression in lung and renal cells.^23^, ^24^ However, whether NADPH oxidase/ROS can lead to LPS‐modulated adipose tissue enlargement via COX‐2 is still not studied. On the contrary, p42/p44 mitogen‐activated protein kinase (MAPK) is reported involving in regulating inflammation and proliferation.^25^, ^26^ In previous studies, we found that activation of p42/p44 MAPK was involved in COX‐2 gene expression in response to various cytokine stimulation.^27^, ^28^ p42/p44 MAPK is also reported as the downstream signalling molecule of ROS, being a possible link between ROS and inflammation‐related diseases.^29^, ^30^ Moreover, studies suggested that activation of p42/p44 MAPK contributes to LPS‐mediated differentiation of human periodontal ligament stem cell.^31^ However, whether ROS‐mediated activation of p42/p44 MAPK contributes to LPS‐induced hyperplasia expansion of adipose tissue remains largely unknown.

In this study, we found that the stimulation of LPS on preadipocytes promoted the expression of COX‐2 gene. In addition, LPS mediated the activation of p42/p44 MAPK via NADPH oxidase‐mediated ROS accumulation. LPS‐regulated preadipocytes proliferation and adipogenesis were attenuated by the inhibition of NADPH oxidase, p42/p44 MAPK and COX‐2. In conclusion, LPS promoted the expansion of adipocytes dependent on COX‐2 gene expression via NADPH oxidase/ROS‐activated p42/p44 MAPK.

## 2 MATERIALS AND METHODS

### 2.1 Materials

Foetal bovine serum (FBS), DMEM medium, TRIZOL and 5‐(and‐6)‐chloromethyl‐2′,7′‐dichlorodihydrofluorescein diacetate, acetyl ester (CM‐H2DCFDA) were purchased from Invitrogen (Carlsbad, CA, USA). Antibodies against COX‐2 were obtained from Santa Cruz Biotechnology (Santa Cruz, CA, USA). PhosphoPlus p47^phox^ were from Assay Biotechnology. PhosphoPlus p42/p44 MAPK antibody and GAPDH were obtained from New England Biolabs (Beverly, MA, USA). *N*‐acetylcysteine (NAC), diphenylene iodonium chloride (DPI), apocynin (APO), U0126, NS398, SC51089, AH6809, L798, 106 and GW627368X were obtained from Biomol. Hybond C membrane, Hyperfilms and enhanced chemiluminescence (ECL) Western blotting detection system were obtained from GE Healthcare Biosciences. XTT assay kit was purchased from Biological Industries. SDS‐PAGE supplies were from MDBio Inc. LPS, Oil red O, enzymes and other chemicals were obtained from Sigma.

### 2.2 Cell culture and adipogenesis

3 T3‐L1 preadipocytes were purchased from Food Industry Research and Development Institute (Hsinchu, Taiwan). Cryopreserved preadipocytes were thawed and cultured in DMEM medium supplemented with 10% FBS at 37°C and 5% CO_2_ atmosphere. Upon reaching confluence, preadipocytes were differentiated to adipocytes by the addition of differentiation cocktail consisting of 0.5 mM methylisobutylxanthine, 1 μg/ml insulin and 0.25 μM dexamethasome into DMEM medium. Preadipocytes were incubated in this differentiation medium (DM)‐I for 48 h at 37°C and 5% CO_2_. Next, DM‐I were changed by DM‐II consisting of DMEM medium with 1 μg/ml insulin, and the cells were further cultured for 2–6 days. Mature adipocytes were identified as Oil Red O‐stained lipid droplets in the cytoplasm.

### 2.3 Oil Red O staining

To verify the successful adipogenesis, at the end of differentiation, the cells were washed with PBS and fixed with 10% formalin for 1 h at room temperature. After fixation, formalin was discarded and the cells were washed with 60% isopropanol once for 5 min. Next, Oil Red O working solution was added to evenly cover the cells for 10 min, followed by the thorough rinse with water. The cells were visualized on a phase‐contrast microscope (DMI 3000 B; Leica), with lipids appeared red. For further lipid quantification in adipocytes, 100% isopropanol was added into Oil Red O and was mixed for 10 min until all Oil Red O was completely dissolved. The mixture of isopropanol and Oil Red O was transferred to 96‐well plate, and the optical density of solution was measured at 490 nm by Epoch™ Multi‐Volume Spectrophotometer System (BioTek). The blank control was designated as 100% isopropanol.

### 2.4 Western blotting

After treating under different experimental conditions, the cells were rapidly washed with ice‐cold PBS, then scraped and collected through centrifugation at 1000 *g* for 10 min. The cell lysates were obtained by lysing the cells with ice‐cold lysis buffer, which further underwent centrifugation at 4500 *g* for 1 h at 4°C to yield the whole‐cell extract. Samples from the supernatant fractions (30 μg protein) were subjected to SDS‐PAGE using 10% running gel for electrophoresis. After that, proteins were transferred to nitrocellulose membranes, followed by the incubation with 5% BSA in Tris‐buffered saline with 0.1% Tween 20 (TTBS) for 1 h at room temperature. Next, the membranes were incubated overnight at 4°C with anti‐COX‐2, anti‐phospho‐p47^phox^, anti‐phospho‐p42/p44 MAPK or anti‐GAPDH antibody. Following the primary antibody incubation, the membranes were incubated with a 1:2000 dilution of anti‐mouse or anti‐rabbit horseradish peroxidase antibody for 1 h at room temperature. Finally, the immunoreactive bands were detected using ECL reagents developed by Hyperfilm‐ECL.

### 2.5 Real‐time polymerase chain reaction (qPCR) and reverse‐transcriptase polymerase chain reaction (RT‐PCR)

Total RNA was extracted from 3 T3‐L1 cells using Trizol and was reversely transcribed into cDNA according to previous study.^23^ The cDNA containing 2 μg of RNA was then used as a template for RT‐PCR with specific oligonucleotide primers for β‐actin and COX‐2 as following: β‐actin: 5′‐GGCATTGTTACCAACTGGGA CGAC‐3′ (sense), 5′‐CCAGAGGCATACAGGGACAGCACAG‐3′ (antisense); COX‐2: 5′‐AAAACCGTGGGGAATGTATGAGC‐3′ (sense), 5′‐GATGGGTGAA GTGCTGGGGAAAG‐3′ (antisense); NOX1: 5′‐TGAACAACAGCACTCACCAA TGCC‐3′ (sense), 5′‐TCATTGTCCCACATTGGTCTCCCA‐3′ (antisense);NOX2: 5′‐ATGGAGGTGGGACAATACA‐3′ (sense), 5′‐CAGACTTGAGAATGGAGGC‐3′ (antisense).^32^; NOX3: 5′‐TTGTGGCACACTTGTTCAACCTGG‐3′ (sense), 5′‐TCA CACGCATACAAGACCACAGGA‐3′ (antisense); NOX4: 5′‐TCATGGATCTTT GCCTCGAGGGTT‐3′ (sense),5′‐AGTGACTCCTCAAATGGGCTTCCA‐3′ (antisense).^33^; EP1: 5′‐GACGATTCCGAAAGACCGCAG‐3′ (sense), 5′‐CAACACC ACCAACACCAGCAG‐3′ (antisense); EP2: 5′‐GATGGCAGAGGAGACGGAC‐3′ (sense), 5′‐ACTGGCACTGGACTGGGTAGA‐3′ (antisense); EP3: 5′‐TGCTGGCTC TGGTGGTGAC‐3′ (sense), 5′‐ACTCCTTCTCCTTTCCCATCTGTG‐3′ (antisense); EP4: 5′‐CTGGTGGTGCTCATCTGCTC‐3′ (sense), 5′‐AGGTGGTGTCTGCTTGG GTC‐3′ (antisense).^34^ The amplification profile consisted of one cycle of initial denaturation at 94°C for 5 min, 30 cycles of denaturation at 94°C for 1 min, primer annealing at 58°C (COX‐2) and 60°C (β‐actin) for 1 min, extension at 72°C for 1 min and one cycle of final extension at 72°C for 5 min. The expression of β‐actin was considered as the internal control.

### 2.6 Cell viability assay

The cells were seeded at a density of 5000 cells/well and then incubated with LPS for 48 h with or without pretreating with specific inhibitor. After that, 50 μl of XTT working solution (prepared according to manufacturer's instruction) was added into each well and the cells were incubated at 37°C and 5% CO_2_ for 2 h. The absorbance was detected at OD450 and OD630 (reference absorbance) by Epoch™ Multi‐Volume Spectrophotometer System (BioTek), thereby determining the cell viability.

### 2.7 Cell number counts

The cells were cultured in 6‐cm dishes and underwent various experimental conditions. After that, the numbers of the cells were counted using HoloMonitor M4 (Phase Holographic Imaging PHI AB, Lund, Sweden) following manufacturer's instruction.

### 2.8 Detection of PEG_2_ release

To detect the release of PGE_2_, 3 T3‐L1 cells were treated with LPS (20 μg/ml) for 0, 6, 16 and 24 h. The levels of PGE_2_ in culture medium were detected with a PGE_2_ ELISA kit (Enzo Life Sciences, Inc.) according to the manufacturer's instructions.

### 2.9 Intracellular ROS detection

DCF‐DA was employed to measure the intracellular ROS levels according to previous work.^23^ 3 T3‐L1 cells were incubated with 1 mM DCF‐DA in PBS for 30 min at 37°C in 5% CO_2_ atmosphere. The medium containing DCF‐DA was removed, and the cells were washed twice with PBS and replenished with 0.5 ml of DMEM medium. Next, the cells were exposed to LPS for the indicated time intervals without or with inhibitor pretreatment for 1 h. After that, the cells were washed twice with ice‐cold PBS and the cell lysates were collected using a lysis buffer (1X PBS containing 20% alcohol and 0.1% Tween 20). The cell lysates were then transferred to 1.5‐ml Eppendorf vials and centrifuged at 10,000 *g* for 1 min at 4°C. As the index of ROS generation, the fluorescence of oxidized DCF‐DA in cell lysates was measured at room temperature by the Infinite 200 PRO multimode reader (Tecan Group). The excitation and emission wavelengths were set at 490 and 530 nm, respectively.

### 2.10 Statistical analysis

The data were shown as the mean ± standard error of the mean (S.E.M.) of at least four independent experiments. Quantitative data were analysed by the GraphPad Prism Program (GraphPad) using one‐way anova followed by Tukey's post hoc test. The *p*‐value at *p* < 0.05 was set as the level of statistical significance.

## 3 RESULTS

### 3.1 LPS stimulated preadipocyte proliferation and COX‐2 gene expression

**FIGURE 1:**
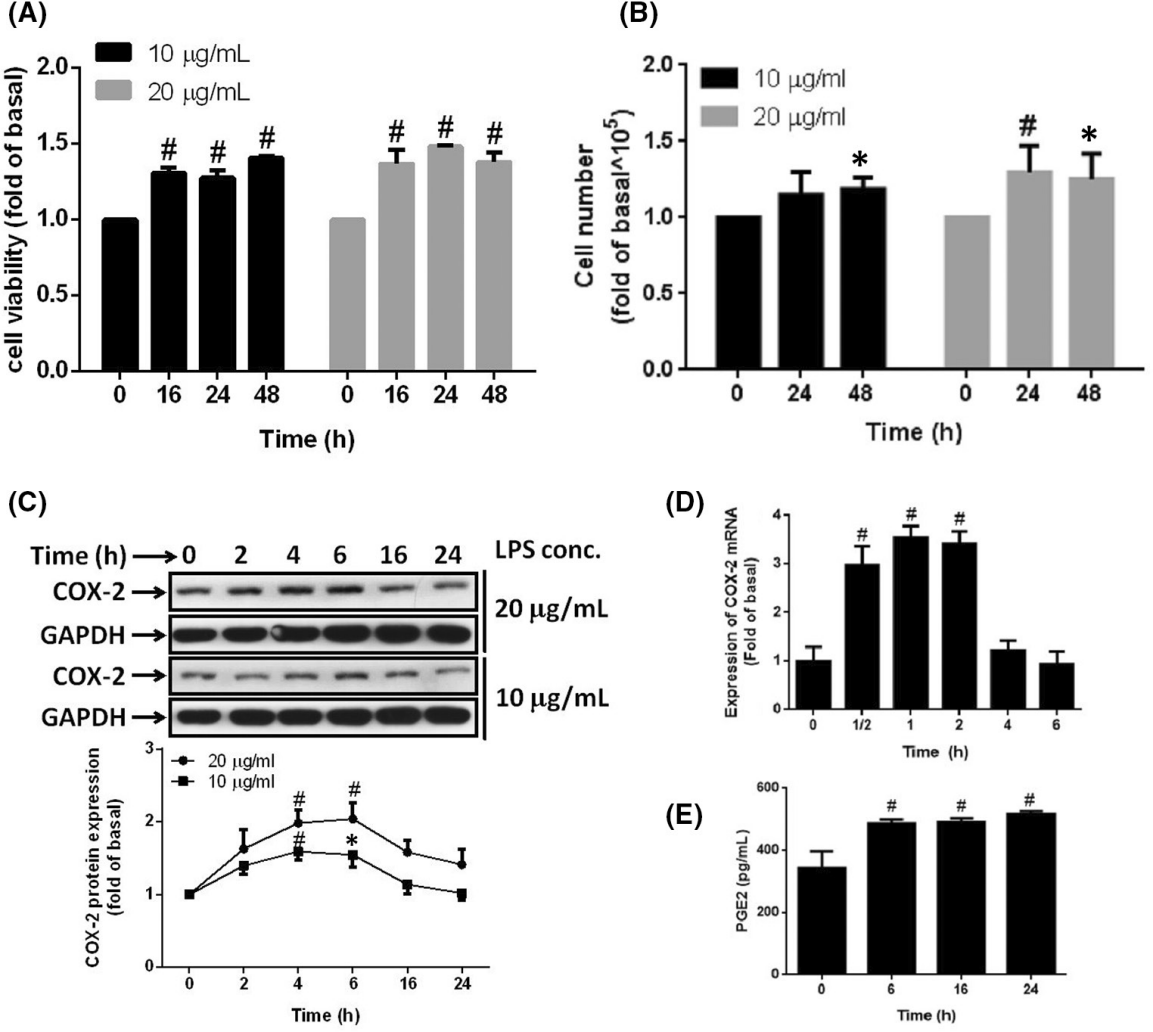
Lipopolysaccharide increased cell proliferation and COX‐2 expression. Serum‐starved 3 T3‐L1 cells were stimulated with 20 or 10 μg/ml of LPS for the indicated time points. At the end of incubation, (A) XTT assay or (B) cell counts were used to evaluate the proliferation of cells. Or cells were harvested and cell lysates or mRNA were extracted. (C) Western blot was used to detect COX‐2 protein expression. (D) qPCR was used to analyse the COX‐2 mRNA expression. (E) PGE_2_ release was detected by ELISA. Data are expressed as means ± SEM of at least four independent experiments (*n* ≥ 4). ^#^ *p* < 0.01, *p < 0.05, as compared with the 0 point group

More and more reports have revealed that the changed microbiota composition in the intestine contribute to obesity.^35^ LPS is the major outer membrane component of Gram‐negative bacteria which are the significant strains in intestinal bacteria.^36^ Once the intestinal epithelium ruptured, bacteria enter tissues and circulate in the whole body.^37^ LPS released by dead bacteria can stimulate peripheral tissues, contributing to the changes of physiological conditions. Here, the stimulation of LPS on preadipocytes, 3 T3‐L1, stimulated the increase of cell viability together with cell numbers in a time‐dependent manner (Figure 1A,B), suggesting that the increase of LPS in adipose tissue promoted the proliferation of preadipocytes correlating to the hyperplasia of adipose tissue. Moreover, we also showed that LPS increased the protein and mRNA expression of COX‐2 in preadipocytes (Figure 1C,D). The expression of COX‐2 resulted in an increase of PGE_2_ release in response to the stimulation of LPS (Figure 1E). This result implicated that inflammation derived from LPS infiltration may contribute to the proliferation of preadipocytes.

### 3.2 Increase of intracellular ROS involved in LPS‐mediated COX‐2 expression and cell proliferation in preadipocytes

**FIGURE 2:**
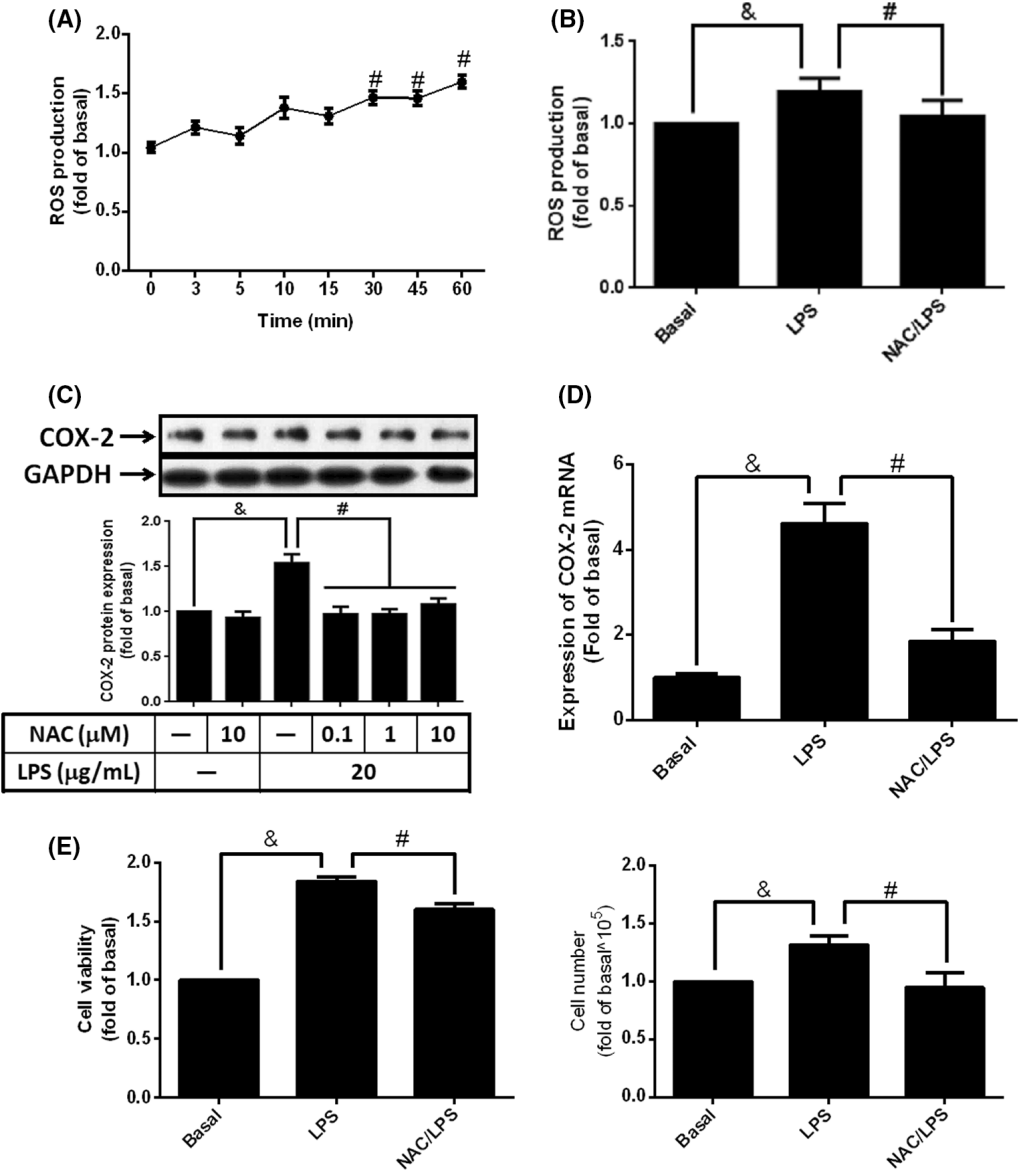
Clearance of intracellular ROS abolished LPS‐mediated COX‐2 expression and cell proliferation. (A) Serum‐starved 3 T3‐L1 cells were stimulated with LPS (20 μg/ml) for the indicated time points; or cells were pretreated without or with 10 μM or different concentrations of NAC for 1 h, and then incubated with 20 μg/ml of LPS for (B) 60 min, (C) 6 h, (D) 2 or (E) 48 h. After treatments, (A and B) intracellular ROS was detected using DCF‐DA probe. (C) COX‐2 protein expression was detected by Western blotting. (D) COX‐2 mRNA expression was analysed using qPCR. (E) The changes of cell proliferation were showed by XTT assay and cell counts. Data are expressed as means ± S.E.M. of at least 4 independent experiments. ^&^ *p* < 0.05, as compared with control group; ^#^ *p* < 0.05, between two indicated groups

Reactive oxygen species are reported to function as signal molecules in response to various stimulators, such as TNF and PDGF.^38^ To evaluate whether LPS modulated inflammation and expansion of preadipocytes via the increase of intracellular ROS, cells were treated with 20 μg/ml of LPS for 0–60 min. The increased intracellular ROS was detected by DCF‐DA fluorescence probe. As shown in Figure 2A, stimulation of LPS promoted the accumulation of intracellular ROS in a time‐dependent manner. Pretreatement of N‐acetylcysteine (NAC), a ROS scavenger, significantly reversed LPS‐regulated ROS accumulation in cytoplasma of 3 T3‐L1 cells (Figure 2B). Furthermore, treatment of NAC significantly attenuated LPS‐stimulated COX‐2 protein and mRNA expression (Figure 2C,D) together with cell proliferation (Figure 2E). Briefly, these results suggested that LPS increased COX‐2 gene expression and preadipocyte proliferation via promoting the increase of intracellular ROS.

### 3.3 LPS‐modulated COX‐2 expression and preadipocyte proliferation via activation of NADPH oxidase

**FIGURE 3:**
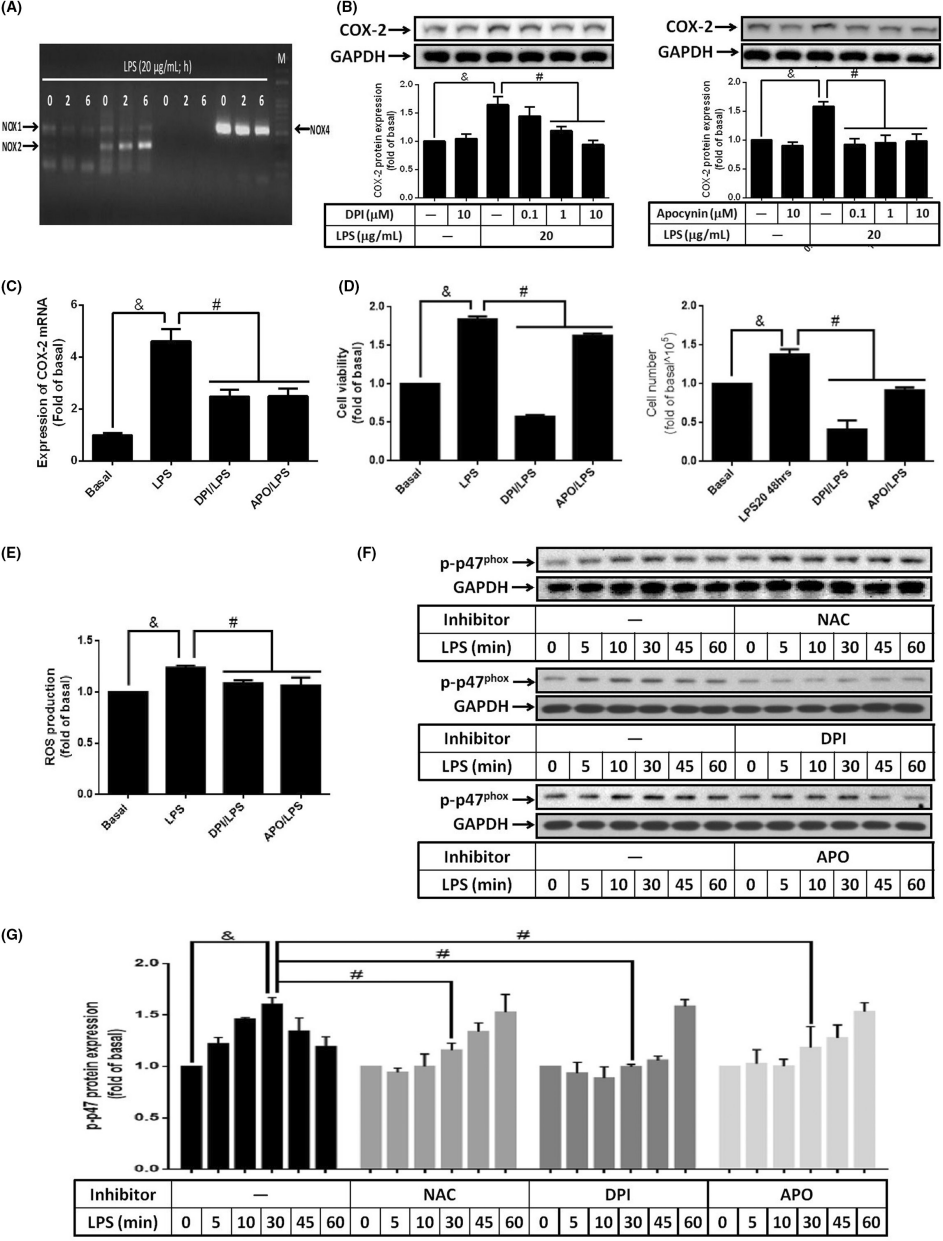
Inhibition of NADPH oxidase reduced LPS‐stimulated COX‐2 expression together with cell proliferation. 3 T3‐L1 cells were pretreated without or with DPI (1 μM or various concentrations), or APO (0.1 μM or various concentrations) for 1 h, then incubated with 20 μg/ml of LPS for (A) 0, 2 or 6 h, (B) 6 h, (C) 2 h, (D) 48 h or (E) 60 min. (F and G) NAC (10 μM), DPI or APO‐pretreated cells were incubated with LPS (20 μg/ml) for the indicated time points. At the end of incubation, (A) EP receptor expression, (B) COX‐2 protein expression or (F and G) phosphorylation of p47^phox^ were detected by Western blot. (C) COX‐2 mRNA expression was analysed by qPCR. (D) The changes of cell proliferation were showed by XTT assay and cell counts. (E) Intracellular ROS was detected by the usage of DCFDA probe. Data are expressed as means ± SEM of at least 4 independent experiments (*n* ≥ 4). ^&^ *p* < 0.05, ^#^ *p* < 0.05, as compared between the two indicated groups

Activation of NADPH oxidase produced ROS and contributed to the imbalance of intracellular oxidative stress.^38^ From the PCR results, 3 T3‐L1 cells expressed NOX1, NOX2 and NOX4 but not NOX3. Stimulation of LPS increased expression of NOX2 but not NOX1 and NOX4 (Figure 3A). To evaluate whether NADPH oxidase involved in LPS‐promoted expression of COX‐2 and cell proliferation, iphenylene iodonium chloride (DPI) or apocynin (APO), inhibitors of NADPH oxidase, were used. Cells were pretreated with various concentrations of DPI or APO for 1 h, and then were stimulated with 20 μg/ml of LPS for 6 h. Western blot was used to detect the expression of COX‐2. We found that LPS‐increased COX‐2 expression was abolished by DPI and APO (Figure 3B). Similarly, expression of COX‐2 mRNA was reduced by APO and DPI in LPS‐treated preadipocytes (Figure 3C). These data implied the participation of NADPH oxidase in LPS‐up‐regulated COX‐2 gene expression of preadipocytes. Moreover, LPS‐promoted increase of cell viability and cell numbers were reversed by both DPI and APO (Figure 3D), suggesting the involvement of NADPH oxidase in LPS‐regulated cell proliferation. Results also showed that treatment of NADPH oxidase inhibitor, DPI or APO, also significantly ameliorated LPS‐regulated intracellular ROS accumulation in preadipocytes (Figure 3E). To evaluate whether LPS activated NADPH oxidase in preadipocytes, cells were pretreated with NAC, DPI or APO for 1 h, then stimulated with LPS for the indicated time intervals. Phosphorylation of p47^phox^, subunit of NADPH oxidase, was detected by Western blot. We found that LPS stimulated the increase of phosphorylated p47^phox^ in a time‐dependent manner, with maximal phosphorylation level at 30 min after LPS treatment (Figure 3F,G). Pretreatment of NAC, DPI or APO retarded the maximal responses of LPS‐regulated p47^phox^ phosphorylation (Figure 3F,G). Taken together, these data indicated that LPS modulated COX‐2 expression and preadipocyte proliferation via NADPH oxidase‐dependent ROS.

### 3.4 Involvement of p42/p44 in LPS‐mediated COX‐2 expression and preadipocyte proliferation

**FIGURE 4:**
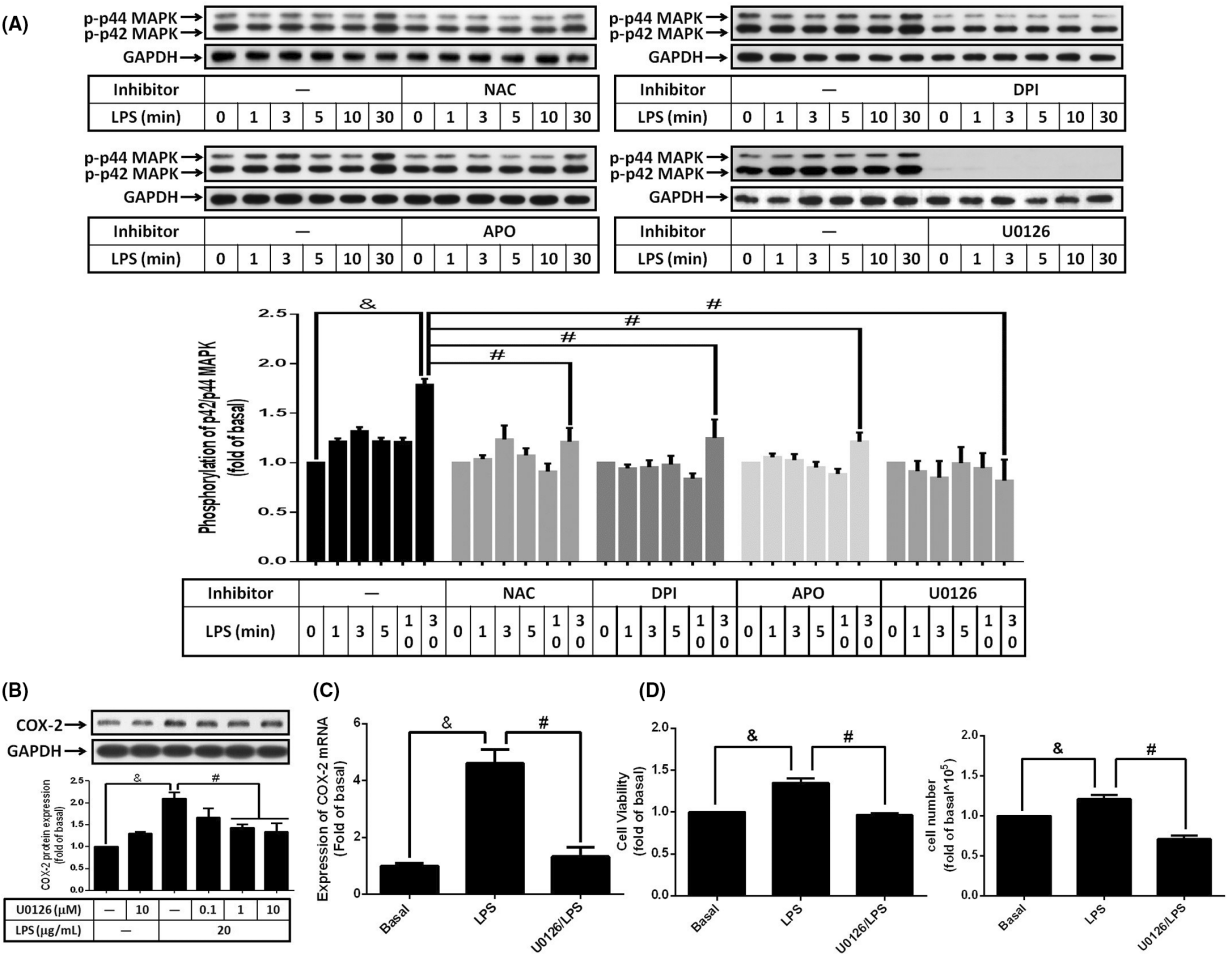
Reduced activation of p42/p44 MAPK abolished LPS‐regulated COX‐2 expression and cell proliferation. 3 T3‐L1 cells were pretreated without or with various inhibitors (indicated concentration) or U0126 (various concentrations or 10 μM) for 1 h, then incubated with 20 μg/ml of LPS for (A) indicated time points, (B) 6 h, (C) 2 h or (D) 48 h. After treatment, (A and B) The phosphorylation of p42/p44 MAPK or COX‐2 protein was detected by Western blotting. (C) COX‐2 mRNA expression was analysed by qPCR. (D) The changes of cell proliferation were showed by XTT assay and cell counts. Data are expressed as means ± S.E.M. of at least 4 independent experiments. ^&^ *p* < 0.05, as compared with control group; ^#^ *p* < 0.05, between two indicated groups

Activation of p42/p44 MAPK is reported in LPS or cytokine‐treated cells^31^ and participated in the regulation of COX‐2 gene expression.^26^ In addition, increase of intracellular ROS is reported as a stimulator modulating p42/p44 MAPK.^39^ To evaluate whether LPS activated p42/p44 MAPK in preadipocytes, cells were treated with LPS for different time intervals, phosphorylation of p42/p44 MAPK was analysed by Western blot. As shown in Figure 4A, stimulation of LPS promoted the increase of p42/p44 MAPK phosphorylation in a time‐dependent manner, which was abolished by U0126, an inhibitor of p42/p44 MAPK upstream protein, MEK1/2, (Figure 4A). Moreover, LPS‐stimulated phosphorylation of p42/p44 MAPK was attenuated by NAC, DPI or APO (Figure 4A), suggesting that NADPH oxidase‐derived ROS may involve in LPS‐mediated p42/p44 MAPK activation. Further experiments showed that pretreatment of U0126 ameliorated LPS‐regulated COX‐2 protein and mRNA expression (Figure 4B,C). Similarly, the increase of preadipocyte proliferation in response to LPS stimulation was improved by the inhibition of p42/p44 MAPK (Figure 4D). Collectively, these data demonstrated that LPS‐promoted COX‐2 expression and preadipocyte proliferation via NADPH oxidase/ROS‐dependent p42/p44 MAPK activation.

### 3.5 COX‐2 contributed to LPS‐increased preadipocyte proliferation

**FIGURE 5:**
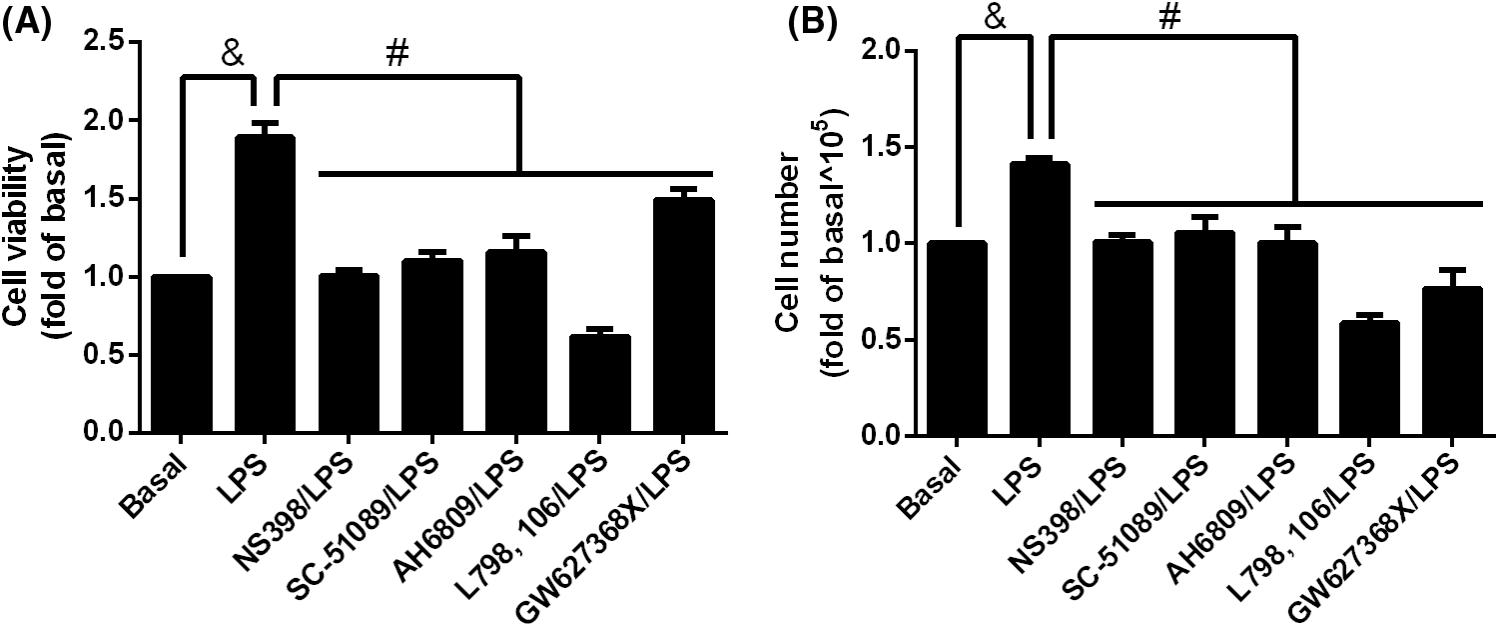
Inhibition of COX‐2 or PGE2 receptors reversed LPS‐induced cell proliferation. 3 T3‐L1 cells were pretreated without or with 10 μM of NS398, SC‐51089, AH6809, L798, 106 or GW627368X for 1 h, then incubated with 20 μg/ml of LPS for 48 h. After incubation, (A) XTT assay was performed and (B) cell numbers were counted. Data are expressed as means ± S.E.M. of at least 4 independent experiments. ^&^ *p* < 0.05, as compared with control group; ^#^ *p* < 0.05, between two indicated groups

To examine the correlation between COX‐2 and cell proliferation, NS398, a selective COX‐2 inhibitor, was used. 3 T3‐L1 cells were pretreated with 10 μM of NS398 for 1 h and then incubated with 20 μg/ml of LPS for 48 h. XTT assay showed that LPS‐increased cell viability was attenuated by the blockage of COX‐2 (Figure 5A). Similarly, cell numbers were reduced in NS398 pretreated groups compared to group of LPS stimulation alone (Figure 5B), revealing that LPS‐modulated preadipocyte proliferation was regulated by COX‐2. The expression of COX‐2 promoted the production of prostaglandin E_2_ (PGE_2_), involving in the regulation of physiological and pathological conditions, such as obesity‐related inflammation.^40^ There are four types of PGE_2_ receptors, EP1, EP2, EP3 and EP4. 3 T3‐L1 cells expressed all four types of PGE_2_ receptors, and stimulation of LPS showed the tendency to increase the expression of EP2 in a time‐dependent manner (Figure S1). To elucidate whether PGE_2_ receptor participated in LPS‐mediated proliferation of preadipocytes, several PGE_2_ receptor inhibitors, SC‐51089 (selective antagonist of EP1), AH6809 (mainly antagonist of EP2), L798, 106 (selective antagonist of EP3) and GW627368X (selective antagonist of EP4), were used to block the function of EP1, EP2, EP3 or EP4, respectively. Result of cell viability assay showed that pretreatment of SC‐51089, AH6809, L798, 106 or GW627368X ameliorated LPS‐increased cell viability (Figure 5A). Similarly, the increase of cell numbers was reversed by SC‐51089, AH6809, L798, 106 or GW627368X in LPS‐treated preadipocytes (Figure 5B). Briefly, these results revealed that COX‐2 contributed to LPS‐upregulated preadipocyte proliferation.

### 3.6 Implication of NADPH oxidase/ROS/COX‐2 cascade in LPS‐mediated adipogenesis

**FIGURE 6:**
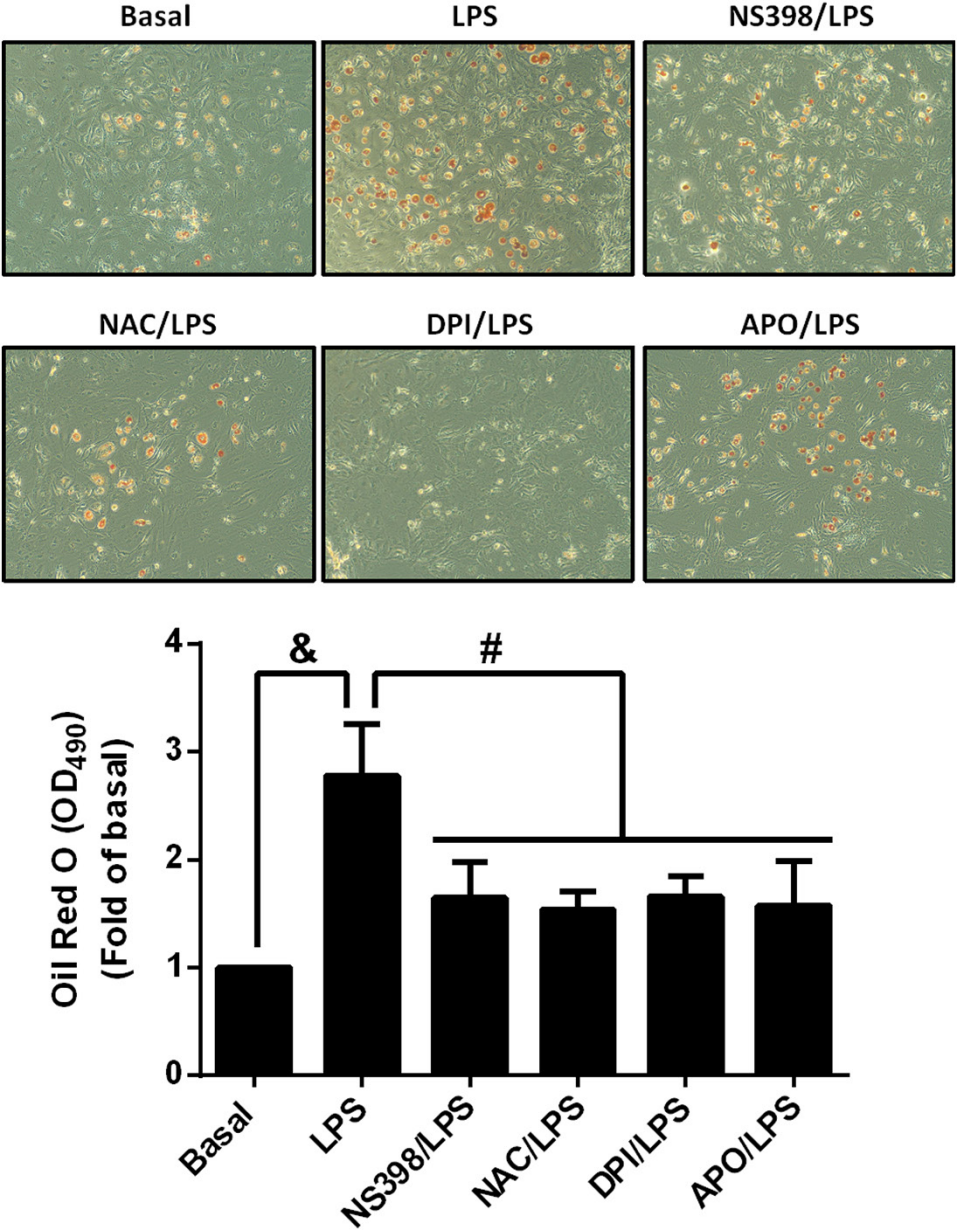
Lipopolysaccharide‐stimulated adipogenesis was attenuated by inhibitors of COX‐2/NADPH oxidase or scavenger of ROS. Cells were pretreated without or with NS398 (10 μM), NAC (10 μM), DPI (0.1 μM) or APO (1 μM) for 1 h, then incubated with 20 μg/ml of LPS for 48 h. After treatment, cells were differentiated by incubated in DM‐I and DM‐II medium. After the process of adipogenesis, the mature adipocytes were stained by Oil red O and the images were captured by microscope. Data are expressed as means ± S.E.M. of at least 4 independent experiments. ^&^ *p* < 0.05, as compared with control group; ^#^ *p* < 0.05, between two indicated groups

To assess whether NADPH oxidase/ROS‐regulated COX‐2 expression participated in LPS‐regulated adipogenesis, preadipocytes were pretreated with the inhibitor or scavenger of NADPH oxidase, ROS and COX‐2, and were then differentiated with the treatment of LPS. At the end of differentiation, adipocytes were identified using Oil red O staining. As shown in Figure 6, stimulation of LPS increased the adipogenesis of preadipocytes and pretreatment of NS398, NAC, DPI or APO alleviated the increase of LPS‐induced effects. Thus, LPS promoted the adipogenesis of preadipocytes via NADOH oxidase/ROS/COX‐2 cascade.

## 4 DISCUSSION

Obesity has been a global health epidemic crisis. Not only adult, children unfortunately are also in this pandemic problem. It is found that more than 41 million children under the age of 5 are in overweight or obese.^1^ Obesity could be developed as early as child age between 2 and 6.^2^ The expansion of fat mass in child age includes both hyperplasia and hypertrophy of adipose tissue, suggesting the importance of proliferation and adipogenesis of preadipocytes.^3^ Moreover, the alterations of the gut microbiota composition, mainly by *Firmicutes*, *Bacteroidetes*, *Actinobacteria* and *Proteobacteria*, correlated to the shape change of our body via skewing body habits and energy metabolism.^1^ With the routine damage of the intestinal epithelium, bacteria or its components, including LPS, may enter the circulation and reach fat tissues. It is reported that scavenger receptor class B type 1 binds both lipids and LPS further provides the routes of LPS translocation.^9^ Here, we reported that stimulation of LPS on preadipocytes increased the proliferation of cells together with the activation of COX‐2 gene. In addition, LPS mediated the activation of p42/p44 MAPK via NADPH oxidase‐mediated ROS accumulation. Pretreatment of the ROS scavenger or inhibitors of NADPH oxidase or p42/p44 MAPK attenuated LPS‐induced COX‐2 expression as well as preadipocyte proliferation. Further data revealed that inhibition of COX‐2 and downstream PGE2 receptors reduced LPS‐modulated preadipocyte proliferation and adipogenesis. Taken together, we found that LPS may enhance preadipocyte proliferation and adipogenesis via NADPH oxidase/ROS/p42/p44 MAPK‐dependent expression of COX‐2.

Cyclooxygenase‐2 plays crucial roles in the production of eicosanoids including PGE_2_. COX‐2 has been reported to regulate inflammation in various pathologies such as cancer and obesity. It has been proved that the activation of COX‐2/PGE2‐EP3 signalling contributes to the development of obesity‐associated adipose tissue inflammation and insulin resistance.^40^, ^41^ Our studies further suggested that expression of COX‐2 may also regulate the proliferation and adipogenesis of preadipocytes which may increase the fat cells during early age obesity. Inhibition of COX‐2 or PGE_2_‐related receptors significantly reduced proliferation and adipogenesis in LPS‐treated preadipocytes, implying the beneficial effects of COX‐2/PGE_2_ receptor inhibitors in avoiding the increase of mature fat cell numbers during controlling child obesity. In fact, it is known that COX‐2 inhibition impairs preadipocyte differentiation but addition of PGE2 bypass the block of COX‐2 inhibitors in adipogenesis.^42^ 3 T3‐L1 cells expressed all four kinds of EP receptors. Stimulation of LPS on preadipocytes showed the tendency to increase the mRNA expression of EP2. Inhibition of four kinds of EP receptors abolished LPS‐mediated preadipocyte proliferation. EP2 may play more roles in mediated LPS‐regulated increased numbers of preadipocytes, but the detail should be more tested in the future. In addition, the potential roles of different EP receptors in obesity were revealed by several studies. High fed diet‐fed EP3(−/−) mice gained more weight and increased epididymal fat mass and adipocyte size relative to EP3(+/+) mice.^43^ PGE_2_/EP4 axis induced by insulin may skew the expression of ATGL and its inhibitor G0S2, resulting in lipolysis and fibrosis of white adipose tissue and ectopic fat deposition.^44^ EP1 may participate in the oxygen consumption and the heat genesis of rat brown adipocytes.^45^ Although the mechanism is unclear, EP2 receptor agonist, ONO‐AE1‐259, improved metabolic disorders via restoration of the subcutaneous adipose tissue in pulmonary emphysema murine model.^46^ It may need more further evaluation for the roles of COX‐2/PGE_2_ axis in the early obesity.

The imbalance of ROS production and antioxidant defences resulted in the accumulation of oxidative stress. Increased oxidative stress via activation of NADPH oxidase or mitochondria may further promote the lipid storage of adipocytes, resulting in hypertrophic expansion.^47^ Moreover, H(2)O,(2) one kind of ROS, accelerates hormonal regimen (IBMX, dexamethasone, and insulin)‐induced adipogenesis of 3 T3‐L1 cells via increasing the expression of peroxisome proliferator‐activated receptor gamma.^48^ Our study showed that LPS increased intracellular accumulation of ROS in preadipocytes via the activation of NADPH oxidase. The activated NADPH oxidase together with increased ROS contributed to cell proliferation and adipogenesis in LPS‐treated 3 T3‐L1 cells. These results were reassembled to what was found in human adipose‐derived stem cells that scavenging ROS production with N‐acetyl‐L‐cysteine attenuated 3‐Isobutyl‐1‐methylanxthine‐induced adipogenesis.^21^ Moreover, treatment with the NADPH oxidase inhibitor can reduce intracellular oxidative stress of adipose tissue and improve fat‐related metabolic syndromes.^49^

In addition to regulating cell proliferation and inflammation,^23^ activation of p42/p44 MAPK has been shown to promote the monocytic and granulocytic differentiation of myeloid cell lines.^50^ Similarly, osteogenic differentiation of human adipose‐derived stem cells is suppressed by treatment of MEK/p42/p44 MAPK inhibitors, PD98059 or U0126.^51^ Here, we reported that stimulation of LPS increased the phosphorylation of p42/p44 MAPK in a ROS‐dependent manner and resulted in COX‐2 expression. Inhibition of p42/p44 MAPK attenuated LPS‐regulated proliferation and adipogenesis of preadipocytes. These results were similar to the effects of leptin that exerts a proadipogenic action in subcutaneous preadipocytes via the activation of p42/p44 MAPK.

In summary, LPS from intestinal microbiota may promote COX‐2 gene expression together with cell proliferation of preadipocytes. Increased phosphorylation of p42/p44 MAPK was regulated by NADPH oxidase‐dependent ROS accumulation in LPS‐stimulated preadipocytes. Treatment with the inhibitors of NADPH oxidase, ROS, p42/p44 MPAK significantly attenuated LPS‐regulated COX‐2 expression and preadipocyte proliferation. Moreover, inhibition of COX‐2 or PGE_2_ receptors reversed LPS effects on proliferation of preadipocytes. LPS‐modulated adipogenesis of preadipocytes was also reduced by the inhibitors of COX‐2 or NAPDH oxidase as well as ROS scavenger. Thus, LPS contributes to hyperplasia of adipose tissues via promoting proliferation and adipogenesis of preadipocytes by NADPH oxidase/ROS/p42/p44 MAPK‐dependent expression of COX‐2 gene. Unveiling the signalling pathways by which LPS is associated with adipose tissue would provide more knowledge for the research and development of preventive and therapeutic strategies of obesity in early life.

## AUTHOR CONTRIBUTIONS

**Chao‐Chien Chang:** Conceptualization (equal); data curation (equal); formal analysis (equal); funding acquisition (equal); writing – original draft (equal); writing – review and editing (equal). **Kee‐Chin Sia:** Conceptualization (supporting); data curation (supporting). **Jia‐Feng Chang:** Writing – original draft (supporting); writing – review and editing (supporting). **Chia‐Mo Lin:** Writing – original draft (supporting). **Chuen‐Mao Yang:** Writing – review and editing (supporting). **I‐Ta Lee:** Writing – review and editing (supporting). **Thi Thuy Tien Vo:** Writing – review and editing (supporting). **Kuo‐Yang Huang:** Writing – review and editing (supporting). **Wei‐Ning Lin:** Conceptualization (lead); data curation (equal); formal analysis (equal); writing – original draft (lead); writing – review and editing (lead).

## CONFLICT OF INTEREST

The authors declare no conflict of interest.

## Supporting information


Figure S1

## Data Availability

Data sharing not applicable.
