# Supplementary figures and images for: Participation of lipopolysaccharide in hyperplasic adipose expansion: Involvement of NADPH oxidase/ROS/p42/p44 MAPK‐dependent Cyclooxygenase‐2
Source: J Cell Mol Med. 2022 Jun 1;26(14):3850–61. doi: 10.1111/jcmm.17419 (PMC9279599; doi:10.1111/jcmm.17419)

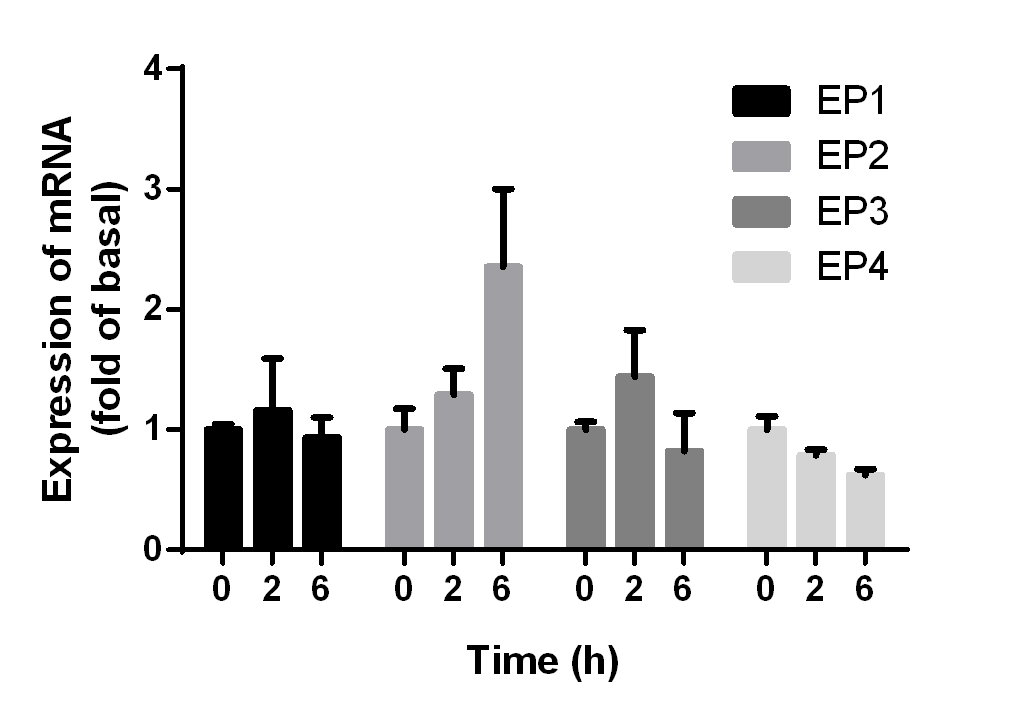

Supplement: Supplementary file 1 — Figure S1 [file JCMM-26-3850-s001.jpg]
